# Independent overexpression of the subunits of translation elongation factor complex eEF1H in human lung cancer

**DOI:** 10.1186/1471-2407-14-913

**Published:** 2014-12-03

**Authors:** Maryna Veremieva, Liudmyla Kapustian, Antonina Khoruzhenko, Valery Zakharychev, Boris Negrutskii, Anna El’skaya

**Affiliations:** State Key Laboratory of Molecular and Cellular Biology, Institute of Molecular Biology and Genetics NASU, 150 Acad.Zabolotnogo Str, Kiev, 03680 Ukraine; Shupyk National Medical Academy of Postgraduate Education, 9 Dorohozhyts’ka str, Kiev, 04112 Ukraine

**Keywords:** Eukaryotic translation elongation factor 1, Macromolecular complexes, Protein biosynthesis, Lung cancer

## Abstract

**Background:**

The constituents of stable multiprotein complexes are known to dissociate from the complex to play independent regulatory roles. The components of translation elongation complex eEF1H (eEF1A, eEF1Bα, eEF1Bβ, eEF1Bγ) were found overexpressed in different cancers. To gain the knowledge about novel cancer-related translational mechanisms we intended to reveal whether eEF1H exists as a single unit or independent subunits in different human cancers.

**Methods:**

The changes in the expression level of every subunit of eEF1H in the human non-small-cell lung cancer tissues were examined. The localization of eEF1H subunits was assessed by immunohistochemistry methods, subcellular fractionation and confocal microscopy. The possibility of the interaction between the subunits was estimated by co-immunoprecipitation.

**Results:**

The level of eEF1Bβ expression was increased more than two-fold in 36%, eEF1Bγ in 28%, eEF1A in 20% and eEF1Bα in 8% of tumor specimens. The cancer-induced alterations in the subunits level were found to be uncoordinated, therefore the increase in the level of at least one subunit of eEF1H was observed in 52% of samples. Nuclear localization of eEF1Bβ in the cancer rather than distal normal looking tissues was found. In cancer tissue, eEF1A and eEF1Bα were not found in nuclei while all four subunits of eEF1H demonstrated both cytoplasmic and nuclear appearance in the lung carcinoma cell line A549. Unexpectedly, in the A549 nuclear fraction eEF1A lost the ability to interact with the eEF1B complex.

**Conclusions:**

The results suggest independent functioning of some fraction of the eEF1H subunits in human tumors. The absence of eEF1A and eEF1B interplay in nuclei of A549 cells is a first evidence for non-translational role of nuclear-localized subunits of eEF1B. We conclude the appearance of the individual eEF1B subunits in tumors is a more general phenomenon than appreciated before and thus is a novel signal of cancer-related changes in translation apparatus.

## Background

Living cells present a significant pool of proteins bound together in stable complexes to perform various biological functions. Forced dissociation of such complexes is important for response of the cell to some extracellular stimuli. Protein synthesis is one of the key milestones in the process of realization of genetic information that includes a great variety of macromolecular complexes. Deregulation of translational control is a critical feature of carcinogenesis [1]. The involvement of translation initiation factors in the cancer progression has been studied extensively [2–5]. Although the elongation factors eEF1A and eEF2 are also involved in the abnormal translation program of cancer cells [6–8], there are very limited data on whether dissociation of the eEF1H complex is possible during the human cancer progression.

The translation elongation complex eEF1H, comprising the eEF1A and eEF1B entities, is involved in the elongation phase of eukaryotic protein synthesis. eEF1A is responsible for the delivery of aminoacyl-tRNA to the A site of ribosome [9, 10]. Eukaryotic eEF1B that consists of the scaffold (eEF1Bγ) and two catalytic (eEF1Bα and eEF1Bβ) subunits, catalyzes GDP/GTP exchange in the eEF1A molecule [11]. Apart from their main role in translation, the eEF1H subunits have been reported to be involved in different processes unrelated to the translational apparatus. In particular, cancer-related overexpression of the eEF1Bβ mRNA was found in lung cancer [12], medulloblastoma [13] and oesophageal carcinoma [14]. Up-regulation of the eEF1Bγ was revealed in breast, colon, gastric and pancreatic tumors [15–18], whereas the eEF1Bα was overexpressed in breast cancer [15]. It remains unclear if the cancer-induced overexpression of the eEF1B subunits is coordinated, or an increase of each subunit occurs independently.

Previously, we have analyzed the eEF1 subunits expression at mRNA and protein levels in the samples of human cardioesophageal and renal carcinomas [19, 20]. The unbalanced expression of eEF1 subunits and the loss of integrity of the eEF1B complex have been observed in human cardioesophageal carcinoma rather than in renal cell carcinoma. That raises the question of whether the cardioesophageal carcinoma case is unique in relation to uncoupled changes in the levels of the eEF1B subunits, or the independent regulation of the subunits expression represents a more common situation in human cancer tissues.

Here, we performed a systematic analysis of the eEF1 subunits expression in 25 samples of human lung carcinoma. Uncoordinated elevation of the eEF1A, eEF1Bβ, eEF1Bγ and, to some extent, eEF1Bα subunits amount was found. An increase in the amount of at least one subunit of the eEF1 complex was found in 52% of the carcinoma specimens. The loss of the eEF1B integrity was confirmed by immunohistochemical analysis which demonstrated specific “cyto-nucleo” distribution of eEF1Bβ in cancer tissue. We have also shown the presence of all eEF1H subunits and the absence of eEF1A-eEF1B interaction in the nuclei of lung adenocarcinoma A549 cells.

## Methods

### Sample tissues

Twenty-five primary tumor specimens and corresponding adjacent normal appearing tissue were obtained from 24 patients with NSCLC (19 adenocarcinomas and 5 squamous lung cell carcinomas) and 1 patient with SCLC during surgery at the Shupyk National Medical Academy of Postgraduate Education (Kiev, Ukraine). The ethical committee of the Institute of Molecular Biology and Genetics NASU (Kiev, Ukraine) has approved the project. All patients who participated in the study signed informed consent forms.

The tissue specimens were immediately immersed in liquid nitrogen and stored under the same conditions. The specimens of tumor and correspondingly paired distal normal appearing tissues were used for mRNA and protein isolation. For immunohistochemistry analysis, the tissue samples were fixed in 10% buffered formalin for 48 h.

### Plasmids and antibodies

The plasmids with eEF1A1, eEF1A2, eEF1Bα, eEF1Bβ and eEF1Bγ cDNAs inserts were kindly provided by C.R. Knudsen, Aarhus University, Denmark, G.M. Janssen, Leiden University, the Netherlands, V.F. Shalak, Institute of Molecular Biology and Genetics NASU, Kiev, Ukraine. The following primary antibodies have been used in experiments: mouse anti-eEF1A (Millipore, USA); in-house mouse anti-eEF1Bα, anti-eEF1Bβ and rabbit anti-eEF1Bγ [20]; mouse anti-eEF1Bα, anti-eEF1Bβ and mouse anti-eEF1Bγ (Abnova, Taiwan); mouse anti-β-actin, (Santa Cruz Biotechnology, USA), rabbit anti-PARP (Cell Signaling, USA), rabbit anti-histone H3.3 and mouse anti-β-Tubulin (Millipore, USA). Immunoblot signals were obtained after incubation with secondary antibodies conjugated with horseradish peroxidase using Immobilon Western Chemiluminescent HRP Substrate (Millipore, USA). Immunofluoresent detection was done with TRITIC – conjugated anti-mouse IgG secondary antibodies (Millipore, USA).

### Northern blot analysis

Total RNA was isolated from frozen tissues using TRI Reagent (Sigma, St Louis, MO, USA). The RNA (10 μg per lane) was electrophoretically separated in 1% agarose gel containing 2.2 M formaldehyde and then transferred to the Nytran Nylon N membrane (Whatman, Maidstone, Kent, UK). Northern blot has been performed as described earlier [19]. To determine whether the eEF1 mRNAs could be up- or down-regulated during carcinogenesis, the β-actin mRNA was used as a reference. No marked tendency of the overall increase or decrease in the actin mRNA level in the lung carcinoma samples was revealed when compared to normal samples.

### Western blot analysis

The frozen tissue specimens were homogenized in the presence of liquid nitrogen in a lysis buffer (10 mM K_2_HPO_4_, 100 mM NaCl, 1% NP-40, 1 mM DTT, 0.1 mM PMSF, pH 7.4), incubated in an ice bath for 30 min and centrifuged at 13000 g for 20 min at 4°C. The protein concentration in the supernatants was determined by the Bradford method [21]. Immunoblotting has been done as described previously [19]. Endogenous β-actin was used as a loading control. The densitometry analysis of signals was performed by the Scion Image program. The target protein expression was evaluated using the relative intensity ratio of target protein/loading control.

More than twofold alterations of the mRNA and protein amount in the tumor samples when compared to the normal ones were taken as a meaningful difference.

### Cell fractionation

A549 cells were cultured in DMEM (Sigma, USA) growth medium with 10% FBS (Sigma, USA) and 1% penicillin/streptomycin (Sigma). Cells were grown up to 7×10^6^ cells/ml and harvested with Trypsin-EDTA. Nuclear fraction was obtained as described in [22] with modifications. Cells were resuspended in 1,5 volume of lysis buffer (10 mM HEPES pH7.9, 1.5 mM MgCl_2_, 0.5% NP-40, 0.2 mM PMSF, 0.5 mM DTT) and incubated on ice for 20 min. Suspended cells were centrifuged at 400 g for 10 min following with supernatant centrifugation at 16000 g for 30 min. Obtained fraction was used as cytoplasmic extract. Pellet after low speed centrifugation was resuspended in 4,5 volumes of sucrose buffer (10 mM HEPES, 0.25 mM sucrose, 1.5 mM MgCl_2_, 10 mM KCl, 0.1% NP-40, 0.5 mM DTT, 0.2 mM PMSF) and incubated on ice for 10 min. Suspension was loaded on sucrose cushion (2 M sucrose) and centrifuged at 400 g for 10 min. After upper buffer and bottom sucrose were discarded, nuclei were resuspended in lysis buffer and centrifuged at 1500 g for 10 min. Procedure was repeated twice. Nuclear pellet was resuspended in 1/2 of starting cell volume of nuclear lysis buffer (20 mM HEPES pH 7.9, 25% glycerol, 0.42 M NaCl, 1.5 mM MgCl_2_, 0.2 mM DTT, 0.2 mM EDTA, 0.2 mM PMSF), incubated on ice for 30 min and centrifuged at 16000 g for 30 min. Obtained fraction was used as source of nuclear protein.

The quality of cytoplasmic and nuclear fractions was analyzed by Western blot with primary rabbit anti-PARP, rabbit anti-Histone 3.3 (nuclear markers) and mouse anti-Tubulin (cytoplasmic marker) antibodies.

### Immunoprecipitation

Cytoplasmic and nuclear extracts from A549 cells were incubated with Protein G Sepharose (Sigma, USA) for 1 hour at 4°C. Rabbit anti-eEF1Bγ antibodies (1.5 μg of antibodies per 1 mg of total protein) were added to pre-cleared lysates and the incubation persisted for 2 hours at 4°C. To precipitate the antibody-protein complex, Protein G Sepharose was added according to the manufacturer’s protocol and incubated for 1 hour at 4°C. To control non-specific binding with Protein G Sepharose, pre-cleared lysate was incubated with the beads only during 1 hour at 4°C. All incubations have been done with orbital shaker. The samples were analyzed by Western blot.

### Immunohistochemistry and immunofluorescence analysis

Cultured A549 cells were fixed with methanol for 5 min at room temperature. Thereafter, the cells were permeabilized with 0.2% Triton X 100 in PBS for 15 min. Non-specific binding was blocked after incubation with Ultra Vision Protein Block buffer (Thermo Scientific, USA) for 5 min. Anti-eEF1Bγ antibodies were applied at dilution 1:100 in PBS with 1% BSA for 1 hour at 37°C. The secondary TRITC conjugated anti-mouse antibodies were applied in dilution 1:100 (Millipore, USA). Cell nuclei were counterstained with Hoechst 33258. Samples were washed and embedded into Mowiol medium (Sigma). Microscopy study was performed using Zeiss LSM 510 META confocal laser scanner microscope (Carl Zeiss, Jena, Germany).

Immunostaining of tissue sections was performed as described earlier [19].

## Results

### Expression of the mRNAs coding for eEF1H subunits in the human lung carcinoma tissue

**Figure 1:**
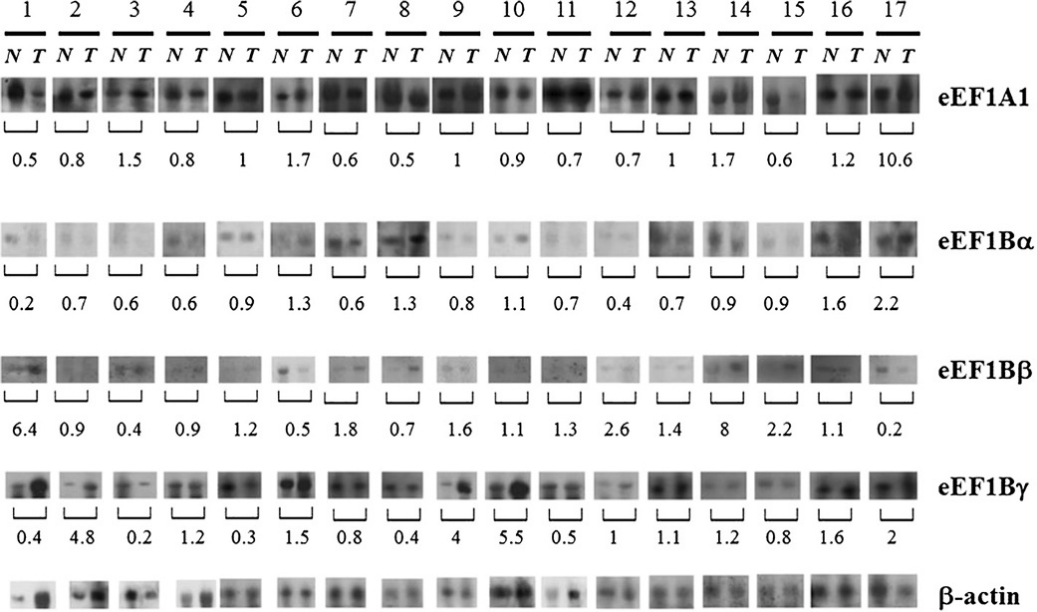
**Comparison of the level of mRNAs coding for different subunits of eEF1H in human lung carcinoma and normal lung.** Representative Northern blots are shown. N – normal issue, T – tumor tissue. Molecular size: eEF1A1 mRNA ~3500 bp, eEF1Bα mRNA ~850 bp, eEF1Bβ mRNA ~1300 bp, eEF1Bγ mRNA ~1500 bp, β-actin mRNA ~1800 bp. Numeration of samples is according to Table 1.

The expression of mRNAs coding for eEF1A1, eEF1Bα, eEF1Bβ and eEF1Bγ in human lung cancer was assessed by Northern blot analysis of 25 clinical tumor specimens. A representative experiment is shown in Figure 1. Analysis of the expression of mRNA coding for the individual eEF1H subunits has shown overexpression of eEF1Bβ mRNA (32% of cases) and eEF1Bγ mRNA (24% of cases) in cancerous samples (Table 1). The expression of mRNA coding for the putative oncogenic isoform eEF1A2 was not detected by Northern blot (data not shown).

**Table 1:** **Changes in the expression levels of the eEF1H mRNAs and corresponding proteins in human lung tumours**

№	Stage	Diagnose	Fold expression, tumor/normal tissues							
			eEF1A		eEF1Bα		eEF1Bβ		eEF1Bγ	
			mRNA	Protein	mRNA	Protein	mRNA	Protein	mRNA	Protein
1	I	SCLC	0.5	0.7	0.2	0.9	6.4	1	0.4	1.2
2	II	Adenocarcinoma	0.8	1.6	0.7	1.3	0.9	1	4.8	2
3	III	Squamous lung cell carcinoma	1.5	1	0.6	1	0.4	1.2	0.2	0.8
4	III	Adenocarcinoma	0.8	1.7	0.6	1.2	0.9	1	1.2	1.9
5	I	Adenocarcinoma	1	3.2	0.9	2.6	1.2	2.6	0.3	2.8
6	II	Adenocarcinoma	1.7	1.7	1.3	0.9	0.5	4	1.5	2.3
7	II	Squamous lung cell carcinoma	0.6	0.9	0.6	1	1.8	1.2	0.8	0.9
8	II	Adenocarcinoma	0.5	1.4	1.3	1	0.7	2.5	0.4	0.7
9	II	Adenocarcinoma	1	1.5	0.8	1	1.6	3	4	0.8
10	II	Adenocarcinoma	0.9	2	1.6	1.2	1.1	2	5.5	2.6
11	II	Adenocarcinoma	0.7	1.2	0.7	1	1.3	0.8	0.5	0.6
12	II	Adenocarcinoma	0.7	1.4	0.4	0.7	2.6	0.7	1	0.9
13	II	Adenocarcinoma	1	2.3	0.7	1.1	1.4	1.8	1.1	1.4
14	I	Adenocarcinoma	1.7	1.1	0.9	1.6	8	1.2	1.2	1.8
15	II	Squamous lung cell carcinoma	0.6	1.6	0.9	0.9	2.2	1.5	0.8	0.3
16	II	Adenocarcinoma	1.2	0.9	1.6	1.3	1.1	1.3	1.6	0.9
17	II	Squamous lung cell carcinoma	10.6	1.6	2.2	3	0.2	1.2	2	0.9
18	II	Adenocarcinoma	1	2	0.9	1.4	1.3	2.4	1.7	1.5
19	II	Adenocarcinoma	0.9	1.8	1	1.4	2.2	2	6	2.3
20	III	Adenocarcinoma	3.3	0.7	2.9	1.3	4.3	0.6	11	0.6
21	II	Adenocarcinoma	0.6	2.1	0.8	1.3	3.2	2.2	1.2	1.3
22	III	Adenocarcinoma	0.8	0.6	0.4	1.5	0.2	1.1	1.6	0.5
23	III	Squamous lung cell carcinoma	0.7	1.4	1.5	1.3	2	2.3	0.8	2.3
24	II	Adenocarcinoma	0.6	0.7	0.5	0.9	1.4	0.3	0.7	17
25	I	Adenocarcinoma	1.1	0.8	0.2	1.2	0.9	0.5	1	0.7

### Expression of protein entities of the eEF1 complex in the human lung carcinoma tissue

**Figure 2:**
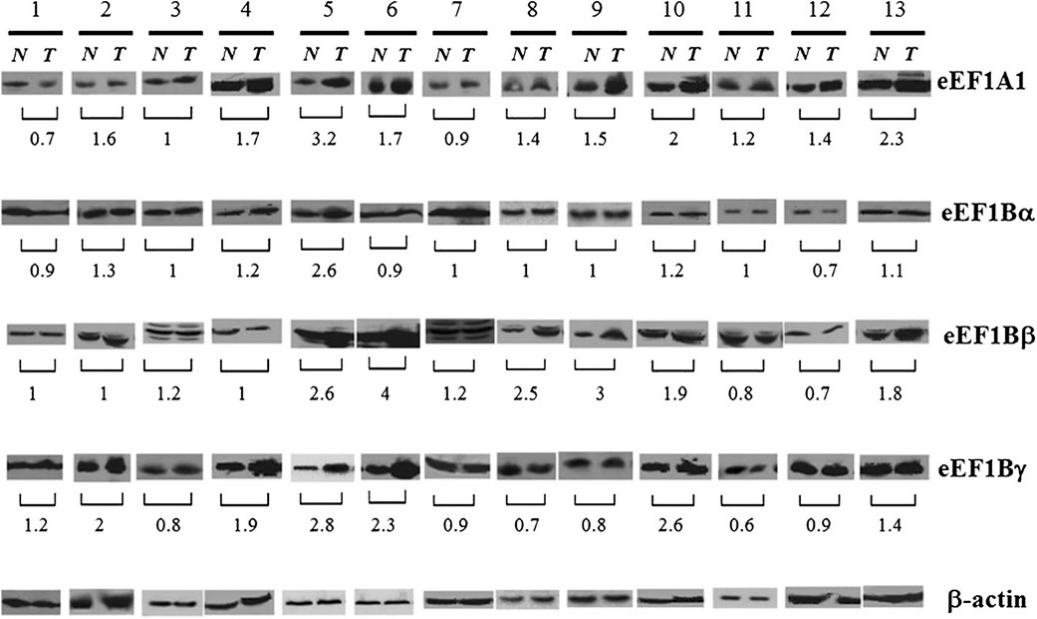
**Comparison of the level of different subunits of eEF1H in human lung carcinoma and normal lung.** Representative Western blots are shown. N – normal tissue, T – tumor tissue. Molecular weights: eEF1A ~50 kDa, eEF1Bα ~28 kDa, eEF1Bβ ~35 kDa, eEF1Bγ ~51 kDa, β-actin ~42 kDa. Numeration of samples is according to Table 1.

The protein content of eEF1 subunits was examined in the same tumor samples by Western blot analysis. A representative experiment is shown in Figure 2. Independent ≥ 2-fold overexpression of at least one eEF1 component was detected in 52% of all tumor specimens (Table 1). A substantial cancer-related elevation was observed for the eEF1Bβ (36%) and eEF1Bγ (28%) subunits. We did not observe any significant changes in the eEF1Bα protein level. In fact, eEF1Bα increased ≥ 2-fold only in two clinical samples out of 25.

Importantly, the unbalanced changes in the eEF1H subunits content were observed in the vast majority of the tumor samples (Table 1). No cancer-induced changes in the expression level of any subunit were found in three patients (the patients’ numbers 4, 7, 16). ≥2-fold overexpression of all eEF1H subunits at protein level in concert was found only in one case (specimen number 5). The coordinated increase in the protein amount of eEF1Bβ and eEF1Bγ was noticed in 5 clinical samples (patients’ numbers 5, 6, 10, 19, 23), i.e. 71% of the specimens, where the overexpression of eEF1Bγ was found. The correlation between overexpression of mRNAs coding for different subunits and the corresponding proteins was observed in nearly half of the tumor specimens where the mRNA overexpression was detected.

### Nuclear localization of eEF1 components in human lung

**Figure 3:**
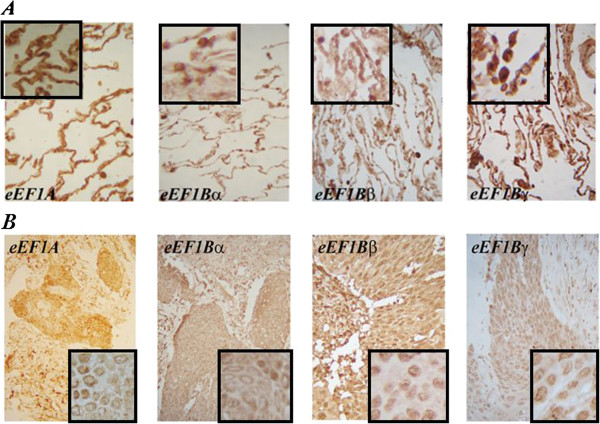
**Immunohistochemical analysis of the eEF1H subunits in human lung carcinoma.** **(A)** Normal tissue, **(B)** Cancerous tissue. The sample 24 (Table 1) was used for analysis. Magnification is 20x, insert – 100x.

Previously, we have observed different locations of the individual eEF1B subunits in the human cardioesophageal carcinoma samples by immunohistochemical analysis [19]. To examine whether the eEF1B subunits demonstrate cancer-related re-localization in human lung cancer the histochemical analysis was performed with the lung cancer samples. Strong staining of all target proteins was detected in both normal and cancerous epithelial cells (Figure 3). Perinuclear appearance of eEF1A and eEF1Bα was observed in the cells of normal and cancerous lung tissues. eEF1Bβ was detected in perinuclear region of normal cells while in the cancerous tissue eEF1Bβ showed both cytoplasmic and nuclear localization. eEF1Bγ was found in cell nuclei and cytoplasm in the normal and cancerous lung tissues (Figure 3). One may speculate that the simultaneous presence of eEF1Bβ and eEF1Bγ in the cancer cell nuclei may be a tumor-related sign of a suggested previously binary eEF1Bβ-eEF1Bγ complex, which could be formed *de novo* or be a remnant of eEF1H.

**Figure 4:**
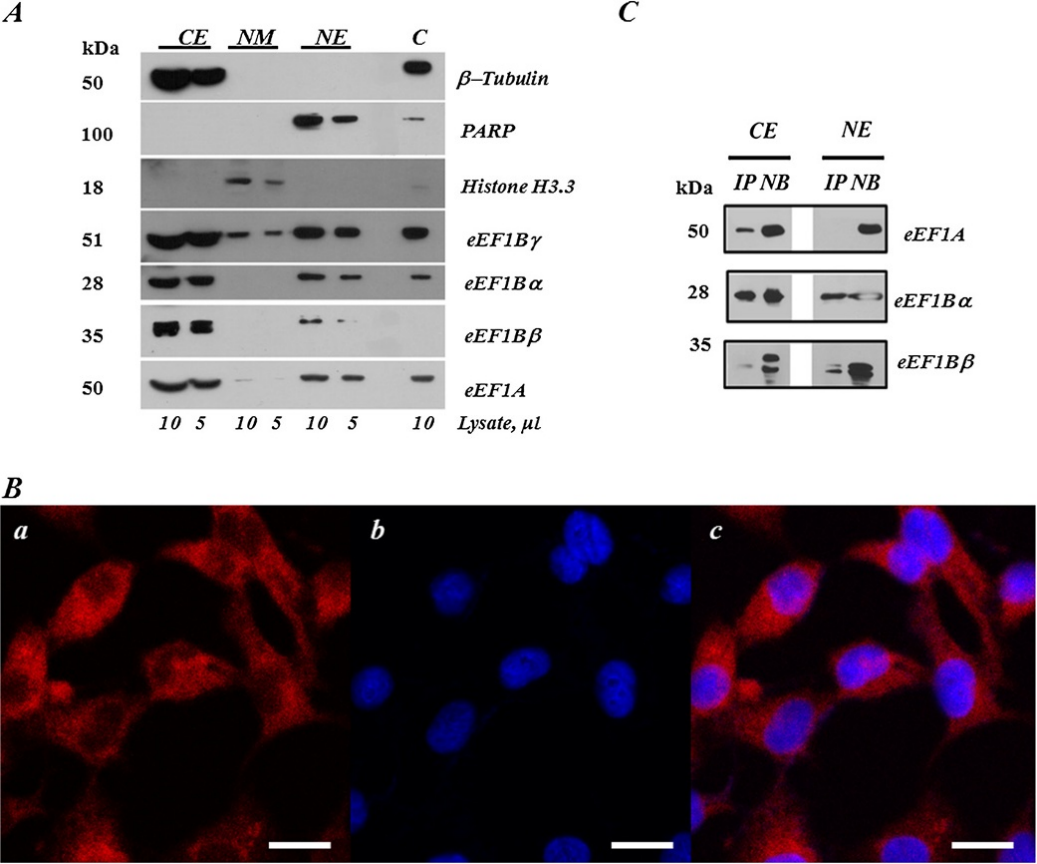
**Distribution of the eEF1H subunits in A549 cells.** **(A)** Subcellular fractionation. CE - cytoplasmic extract, NM – nuclear membrane fraction, NE – nuclear extract, C — control (the pellet after taking out the cytoplasmic fraction and before isolation of nuclei). **(B)** Immunofluorescent analysis of eEF1Bγ localization. (a) Localization of eEF1Bγ in A549 cells, (b) nuclear staining with Hoechst 33342, (c) merge. Scale bar 20 μm. **(C)** Co-immunoprecipitation of eEF1A and eEF1Bγ. CE – cytoplasmic extract, NE – nuclear extract. IP – fraction of immunoprecipitated proteins, B – fraction of non-bound proteins.

To analyze nuclear localization of the eEF1Bβ and eEF1Bγ subunits in more detail, we isolated the fraction of nuclear proteins from the human lung adenocarcinoma cell line A549. Unexpectedly, all eEF1H subunits were found to be present in both cytoplasmic and nuclear fractions of A549 cells (Figure 4A) which contradicted the data on the cancer tissues where eEF1A and eEF1Bα were not found in nucleus. The nucleus-located eEF1A and eEF1Bα did not seem to be contaminants from outer side of nuclear membrane, as these subunits were not present in the nuclear membrane fraction of A549 cells (Figure 4A). eEF1Bγ was found in all subcellular fractions including the nuclear membrane fraction, indicating the possibility of cross-contamination of the fractions with eEF1Bγ. Therefore, a confocal microscopy of A549 cells was conducted to specify intracellular localization of eEF1Bγ. As seen in Figure 4B, the eEF1Bγ subunit was present in both cytoplasm and nucleus of A549 cells. Thus, in the lung cancer cell line all subunits of eEF1B demonstrate both cytoplasmic and nuclear localization.

The question remains whether the nucleus-localized subunits of eEF1B can interact with each other in A549 cells. Since eEF1Bγ is believed to serve as a core subunit for the eEF1B complex, we used anti-eEF1Bγ antibodies to co-precipitate the eEF1Bα and eEF1Bβ subunits from both cytoplasmic and nuclear fractions of the A549 cell line (Figure 4C). Co-precipitation of all eEF1B subunits in the nuclear extract was revealed, suggesting that the eEF1B complex may be present in the nucleus. Unexpectedly, in the nuclear fraction of A549 cells the interaction of the eEF1B complex with eEF1A was not detected despite eEF1A being present in the nuclear fraction. Contrary to that, the eEF1A-eEF1B interaction was observed in the cytoplasmic extract (Figure 4C).

## Discussion

The lack of correlated changes in the expression level of all eEF1H subunits suggests the presence of the individual, non-complexed subunits of eEF1H in lung cancer tissue. Oncogenic properties of eEF1Bβ were described earlier [23]. In particular, overexpression of eEF1Bβ inhibits E3 ubiquitin ligase SIAH- 1, the enzyme which can suppress tumor development via targeting of oncogenic and anti-apoptotic proteins [24, 25]. The role of individual eEF1Bγ in cancer is not so clear. This subunit is a participant of oxidative stress response pathway. In the absence of eEF1Bγ this pathway is constitutively active [26], thus, overexpression of individual eEF1Bγ in tumor cells may impair cellular ROS detoxification, which sensitizes tumor cells to ROS-induced cell death. Recently, it has been shown that eEF1Bγ, being phosphorylated at Ser294 by DOA protein kinase, down-regulates transport of several classes of membrane organelles, including peroxisomes and mitochondria, along microtubules [27]. An effect of this phosphorylation on the ability of eEF1Bγ to be in complex with other subunits of eEF1B remains to be investigated. Recently, ectopically expressed individual eEF1Bγ was shown to interact with NFκB and positively regulate NFκB-mediated signal cascades [28]. NFκB is known factor of cancer-related inflammation which is widely involved in the initiation and progression of cancer.

**Figure 5:**
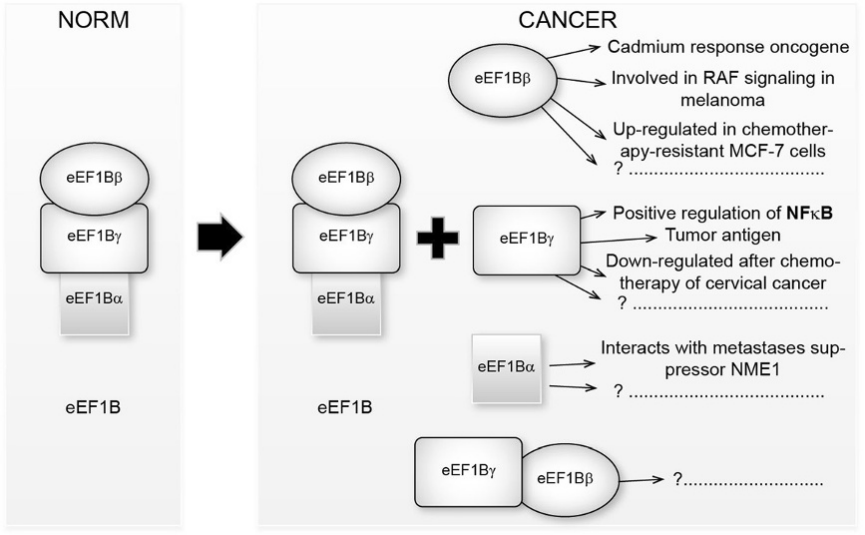
**Cancer**-**induced changes in the composition of the eEF1H complex.** In cancer cells, both eEF1B and its individual subunits are present, as well as hypothetic eEF1Bβ-eEF1Bγ complex. eEF1Bβ and eEF1Bγ may contribute to the cancer development while a role of individual eEF1Bα is unknown.

The functions of a protein may depend on whether or not this protein is present in a cell as a single entity or as a component of high-molecular weight complex [29]. Our hypothesis is that the eEF1B complex functions in the same way both in tumor and in normal tissues, providing nascent polypeptide elongation process with the GTP-bound form of eEF1A while free subunits of eEF1B are directed towards fulfillment of different cancer-related duties (Figure 5).

It is noteworthy that more than 2-fold elevation of eEF1Bβ protein in the cancer tissues relative to the control has occurred in 71% of the specimens which demonstrate the eEF1Bγ overexpression (Table 2). Similar coordinated increase of eEF1Bβ and eEF1Bγ subunits content was observed previously in human cardioesophageal carcinoma [19]. Parallel increase in the eEF1Bβ and eEF1Bγ amount in cancer tissues hints that these subunits of eEF1B may act together under cancer conditions. eEF1Bβ plays a role as a guanine exchange factor, while eEF1Bγ may serve as an anchoring unit for eEF1Bβ, providing specific subcellular localization of the latter. Importantly, the literature data suggest that both eEF1Bβ and eEF1Bγ possess pro-tumor properties [30–35] while eEF1Bα does not (Figure 5).

**Table 2:** **Overexpression of eEF1H subunits at protein level in different human cancer tissues**

eEF1 subunit	% of cases		
	Cardioesophageal carcinoma [19]	Lung carcinoma	Renal carcinoma [20]
eEF1A	36	20	5
eEF1Bα	20	8	0
eEF1Bβ	36	36	5
eEF1Bγ	36	28	5
All subunits	72	52	16

Subcellular localization of the eEF1H subunits was investigated in the lung cancer tissue samples and cell line. All eEF1H subunits were found in nuclei of A549 cells (Figure 4A), whereas the only eEF1Bγ and eEF1Bβ nuclear localization was found in the lung cancer specimens by immunohistochemical investigation (Figure 3). This difference may be patient-specific or represent a genuine divergence in the eEF1B localization between cultivating cells and cancer tissues. More importantly, the immunoprecipitation analysis did not reveal the classic eEF1H complex in nucleus, as the eEF1A-eEF1B interaction was not detected there despite the presence of all factors in the nuclear fraction. Molecular reasons for this are under investigation and may be related to a different level of post-translational modifications of the eEF1A or eEF1B components. Notably, the phosphorylation of eEF1Bβ can influence its interaction with eEF1A [36]. A reason for the presence of the eEF1H subunits in nucleus is unknown. The data on the existence of nuclear protein synthesis remain controversial [36–43]. As eEF1A and eEF1B did not interact in the nuclear fraction of the cancer cells, this argues against nuclear translation and suggests independent roles of eEF1A and eEF1B. eEF1B was reported to participate in the cell cycle events [44], interact with NFκB [28], play an essential role in the oxidative stress response pathway [26, 45], contribute to cytoskeleton rearrangements [46] and proteolytic system [47].

The analysis of data described here and in [19] uncovered an interesting trend related to overexpression of the eEF1H subunits in different human cancers (Table 2). Cardioesophageal and lung carcinomas showed increased level of expression of all eEF1H subunits observed in 8-36% of all samples, depending on the subunit and tumour type. However, as the changes in the subunits expression are not coordinated, the elevated protein levels of one or another subunit expression were found in 72% of cardioesophageal [19] and 52% of lung carcinomas. Interestingly, we did not observe marked difference in the eEF1H proteins expression in human kidney tumours [20] where the overexpression of the eEF1A and eEF1Bγ proteins was found in two diverse tumour samples out from eighteen specimens researched. This finding favours the specificity of the phenomenon and suggests that the combined increase of different eEF1B subunits of the eEF1H complex may be considered as useful addition to the panel of markers for lung and cardioesophageal carcinomas.

## Conclusions

Taken together, our data revealed that structurally and functionally independent existence of the subunits of the translation elongation complex eEF1B in tumour tissues may be a widespread, albeit specific phenomenon. This finding provides new insights into understanding of principles of the organization of translation apparatus in cancer cells and contributes to the panel of potential markers for lung and cardioesophageal carcinomas.

## Abbreviations

eEF1H: “heavy” form of translation elongation factors complex
eEF1A: subunit A of eEF1H
eEF1B: sub-complex of eEF1H which consists from eEF1Bα, eEF1Bβ and eEF1Bγ subunits
SCLC: small cell lung carcinoma.
